# Evaluation of diagnostic performance of rK28 ELISA using urine for diagnosis of visceral leishmaniasis

**DOI:** 10.1186/s13071-016-1667-2

**Published:** 2016-07-04

**Authors:** Prakash Ghosh, Khondaker R. H. Bhaskar, Faria Hossain, Md Anik Ashfaq Khan, Aarthy C. Vallur, Malcolm S. Duthie, Shinjiro Hamano, Md Abdus Salam, M. Mamun Huda, Md Gulam Musawwir Khan, Rhea N. Coler, Steven G. Reed, Dinesh Mondal

**Affiliations:** Laboratory Sciences Division, International Center for Diarrhoeal Diseases Research, Dhaka, Bangladesh; Department of Biochemistry, University of Utah, Salt Lake City, USA; Infectious Disease Research Institute, Seattle, USA; Department of Parasitology, Institute of Tropical Medicine Nagasaki University, Nagasaki, Japan; Global COE programme, Nagasaki University, Nagasaki, Japan; Department of Basic Medical Sciences, College of Medicine, King Saud bin Abdulaziz University for Health Sciences, Jeddah, KSA Saudi Arabia; Department of Pediatrics Immunology Division Faculty of Medicine and Health Science, University of Sherbrooke, Sherbrooke, QC Canada

**Keywords:** Visceral leishmaniasis, Diagnosis, rK28, rK39, rKRP42, ELISA, Serum, Urine, Bangladesh

## Abstract

**Background:**

Recombinant fusion proteins are now commonly used to detect circulating antibodies for the serodiagnosis of visceral leishmaniasis (VL) in Asia, Africa and the Americas. Although simple, these tests still require blood collection and their use in remote settings can be limited due to the need of collection devices, serum fractionation instrument and generation of biohazardous waste. The development of an accurate and non-invasive diagnostic algorithm for VL, such as could be achieved with urine, is desirable.

**Methods:**

We enrolled 87 VL patients and 81 non-VL individuals, including 33 healthy endemic controls, 16 healthy non-endemic controls, 16 disease controls and 16 tuberculosis (TB) patients. We compared the efficacy of recombinant antigens rK28, rK39 and rKRP42 for the diagnosis of VL when either serum or urine were used to develop antibody-detection ELISA.

**Results:**

As expected, each of the antigens readily detected antibodies in the serum of VL patients. rK28 ELISA showed the highest sensitivity (98.9 %), followed by rK39 and rKRP42 ELISA (97.7 and 94.4 %, respectively); overall specificity was > 96 %. When urine was used as the test analyte, only a marginal drop in sensitivity was observed, with rK28 ELISA again demonstrating the greatest sensitivity (95.4 %), followed by rK39 and rKRP42 ELISA, respectively. Again, the overall specificity was > 96 %.

**Conclusions:**

Our data indicate the potential for using urine in the diagnosis of VL. Detection of antibodies against rK28 demonstrated the greatest sensitivity. Together, our results indicate that rK28-based antibody detection tests using urine could provide a completely non-invasive tool amenable for diagnosis of VL in remote locations.

**Electronic supplementary material:**

The online version of this article (doi:10.1186/s13071-016-1667-2) contains supplementary material, which is available to authorized users.

## Background

Visceral leishmaniasis (VL), also known as kala-azar, is a vector-borne systemic disease caused by infection with obligate intracellular parasites of the *Leishmania donovani* complex. The disease is closely associated with poverty and socio-economic factors, and can be fatal if left untreated [1, 2]. Bangladesh, India, Nepal, South Sudan, Sudan and Brazil account for approximately 90 % of the annual 500,000 incidences worldwide [3, 4]. However, the burden of VL in the Indian sub-continent (Bangladesh, India, Nepal) has been reduced significantly. Thanks to the efforts of the kala-azar elimination program (KEP) which was initiated in 2005 with the aim to eliminate the disease as a public health problem [5]. To continue this trend and streamline the elimination program, a combined strategy of early case detection, treatment and integrated vector control is needed [6]. As per the strategy of the programme, the consolidation phase of this elimination programme is aiming to restrict the propagation of VL by employing active case detection strategy in endemic areas [7].

The definitive diagnosis of VL is direct observation of *L. donovani* in spleen, bone-marrow or lymph-node aspirate. However, the use of direct detection methods in field settings is precluded by numerous factors, including the risk of potential hemorrhage, the need for trained personnel and the need for a reference clinic. Most molecular methods also require technological expertise and laboratory equipment, and are therefore expensive. Several serology-based methods, like ELISA with crude or recombinant antigens, indirect fluorescent antibody test (IFAT), western blot and direct agglutination test (DAT) have provided good diagnostic performance [1, 8]. Among them, DAT has been a widely used technique in the laboratory as well as in field settings but this method is cumbersome to perform, needs trained personnel and sometimes gives ambiguous results [1, 8, 9]. The rK39 recombinant antigen, which is derived as a part from a *L. chagasi* kinesin-related gene, has become widely used to detect serum antibodies in a rapid diagnostic test (RDT) format to diagnose VL at the point of care [1]. To overcome the reduced sensitivity observed for rK39 RDT in other VL-endemic areas such as Africa relative to the Indian sub-continent [10, 11], the rK28 recombinant antigen was developed by fusing three proteins (*L. donovani* haspb1, *L. donovani* haspb2 and *L. donovani* kinesin) and has presented promising sensitivity and specificity when evaluated on serum samples from Bangladesh and Sudan [12]. Another recombinant kinesin-related protein derived from *L. donovani*, rKRP42, has also been reported with an excellent performance for diagnosis of VL [13, 14].

Although popular, most dipstick tests have operational limitations, because according to typical manufacturer provisions, they should be performed using serum. This requirement necessitates blood collection and fractionation, but the use of centrifuges or microfuges is not common at the point of care [15]. In addition, children often refuse to give blood.

To overcome the current limitations of diagnostic methods, an alternative strategy is desirable that should have several characteristics including non-invasive, accurate and feasible. Several non-invasive methods, assessing either saliva, oral fluid, buccal swab or urine, have been reported with promising diagnostic performance for VL [16–19], including a study reporting excellent diagnostic performance of urine ELISA with an rKRP42 recombinant antigen [20]. Several recent studies in India and Bangladesh reported similarly satisfactory performance of rK39 RDT using urine [17, 21]. Although rK28 is indicated as an excellent diagnostic antigen capable of detecting antibodies in serum, to date there have been no reports evaluating its diagnostic value using urine. In this study, we therefore compared the sensitivity and specificity of rK28, rK39 and rKRP42 ELISA developed with either serum or urine. Our data indicate an excellent diagnostic efficacy of rK28 ELISA developed with urine and suggest that rK28 RDT could be used as a completely non-invasive test at the point of care.

## Methods

### Study sites

This study was conducted in two sites: Rajshahi Medical College Hospital (RMCH), Bangladesh and the International Centre for Diarrheal Disease Research, Bangladesh (icddr,b). TB patients included in this study were from National TB and Chest Hospital, Mohakhali. This study was approved by the icddr,b and Rajshahi Medical College ethical review committees. Informed written consent was collected for each participant or the legal guardian for children.

### Study population

A total of 168 participants were enrolled within the period June 2013 - August 2014; of these, 87 were VL patients and 81 non-VL individuals. All VL patients had active VL disease, did not have a previous history and were diagnosed either by detecting parasites in spleen smear or according to the kala-azar national guidelines [7]. All of the enrolled VL patients were followed up for 6 months after treatment for cure assessment. The non-VL group consisted of 33 healthy endemic controls (EC), 16 healthy non-endemic controls (NEC), 16 disease controls (DC) and 16 TB patients. The participants of the EC group were inhabitants living in kala-azar endemic area with no previous history of kala-azar and all of them were negative for rK39 dipstick test. The participants of the NEC group were from a non-endemic area and reported no history of travel to a VL endemic area before participating in this study. All of the EC and NEC were also observed by the physician and annulled of any signs and symptoms of any other disease before enrolment. All of the TB patients were confirmed by being positive in acid fast bacilli microscopy. The participants of the DC group were diagnosed as infectious diseases other than TB and were admitted in RMCH (Additional file 1: Table S1).

### Blood and urine collection

A volume of 3.0 ml venous blood was collected from each participant in an EDTA-free sterile tube (BD tube), then serum was separated by centrifugation and stored at -20 °C. A volume of 5.0 ml urine was collected from each participant into a tube containing a preservative (0.1 % NaN_3_), then stored at 4 °C. All serum and urine samples were transported from Rajshahi Medical College Hospital to icddr,b with maintenance of the cold chain. After arrival at icddr,b serum and urine samples were stored at -20 °C and at 4 °C, respectively. All laboratory methods were performed within 6 months of sample collection.

### Laboratory methods: rK39, rK28 and rKRP42 ELISA

Antibody capture ELISA were performed according to the methods described in previous studies [12, 14, 22]. In brief, flat bottom 96-well microtiter plates (Greiner Bio-one GmbH, Frickenhausen, Germany) were coated with rK39, rK28 and rKRP42 antigens in PBS, 25 ng/well for serum and 50 ng/well for urine (pH 7.4) and were incubated overnight at 4 °C. On the following day, plates were gently washed three times with wash buffer (PBS containing 0.05 % Tween 20; pH 7.4) and then incubated with blocking buffer (PBS containing 1 % BSA; pH 7.4) for 3 h at 37 °C to avoid any non-specific reactivity. After washing five times with wash buffer, 50 μl of 1:400 times diluted serum samples in dilution fluid (DF: PBS containing 0.1 % BSA and 0.05 % Tween 20) were added to each well and incubated for 1 h at 37 °C. For the urine samples, two different dilutions (1:2 and 1:10) along with undiluted urine were tested to validate the cut-off. Finally, two times diluted urine in DF was applied to the respective wells and then incubated for 1 h at 37 °C. After incubation, the plates were washed five times with wash buffer, and then 100 μl of peroxidase-conjugated rabbit anti-human IgG (Jackson Immnuoresearch, PA, USA) (1:5000 times dilution in DF) was added to each well and incubated for 1 h at 37 °C. After washing five times, 100 μl of TMB substrate (Sigma- Aldrich, Missouri, USA) was added to each well then the plate was placed in the dark for 20 min. The reaction was finally stopped by the addition of 50 μl of 1 N H_2_SO_4_. The optical density (OD) was measured at 450 nm in a micro-plate reader (Biotek).

### Statistical analyses

A receiver-operator characteristic (ROC) was constructed for each ELISA to determine all possible combinations of sensitivity and specificity, and an optimal cut-off was taken that clearly differentiated between VL and non-VL subjects at 95 % confidence intervals. All types of controls were used together as controls to construct the ROC curve. The ROC curve was constructed using the SPSS software (version 20). Sensitivity and specificity with 95 % CI were calculated using exact binomial methods for proportions. The value of the area under curve obtained from ROC curve analysis regarded as the metric to evaluate the diagnostic accuracy of each antigen in this study [12]. Cohen’s kappa coefficient (k) was determined for agreement testing between different ELISA. The values of Cohen’s k coefficients were interpreted according to Landis & Koch [15]: 1.00–0.81: excellent; 0.80–0.61: good; 0.60–0.41: moderate; 0.40–0.21: weak; and 0.20–0.00: negligible agreement.

## Results

### Participants’ characteristics

In the VL group, 64.4 % of participants were male and 35.6 % were female; in the non-VL groups, 69.1 % of participants were male and 30.9 % were female. The ratio adult to child for VL patients was 1:1.5, but only three children were recruited across the control groups. The average duration of fever in VL, DC and TB group was comparable. All of the VL patients presented splenomegaly, while 67.8 % of VL patients’ displayed hepatomegaly (Table 1).

**Table 1:** Participant data

Group	VL	DC	EC	TB	NEC
Age (years, mean ± SD)	23.09 ± 14.20	33.62 ± 18.98	31.60 ± 8.71	38.93 ± 14.49	22.93 ± 9.49
Children (*n*)	35	3	0	0	0
Adult (*n*)	52	13	33	16	16
Sex					
Male (*n*)	56	10	25	10	11
Female (*n*)	31	6	8	6	5
Splenomegaly (*n*)	87	5	–	–	–
Duration of fever (mean ± SD)	20.36 ± 13.31	17.43 ± 12.19	–	22.93 ± 9.49	–
ESR (mean ± SD)	96.29 ± 35.17	93.43 ± 35.67	–	106.31 ± 19.14	–
Hepatomegaly (*n*)	59	1	–	–	–
Blackening (*n*)	84	–	–	–	–
Pancytopenia (*n*)	31	–	–	–	–

### Sero-diagnostic performance of rK39, rK28 and rKRP42 antigens

**Fig. 1:**
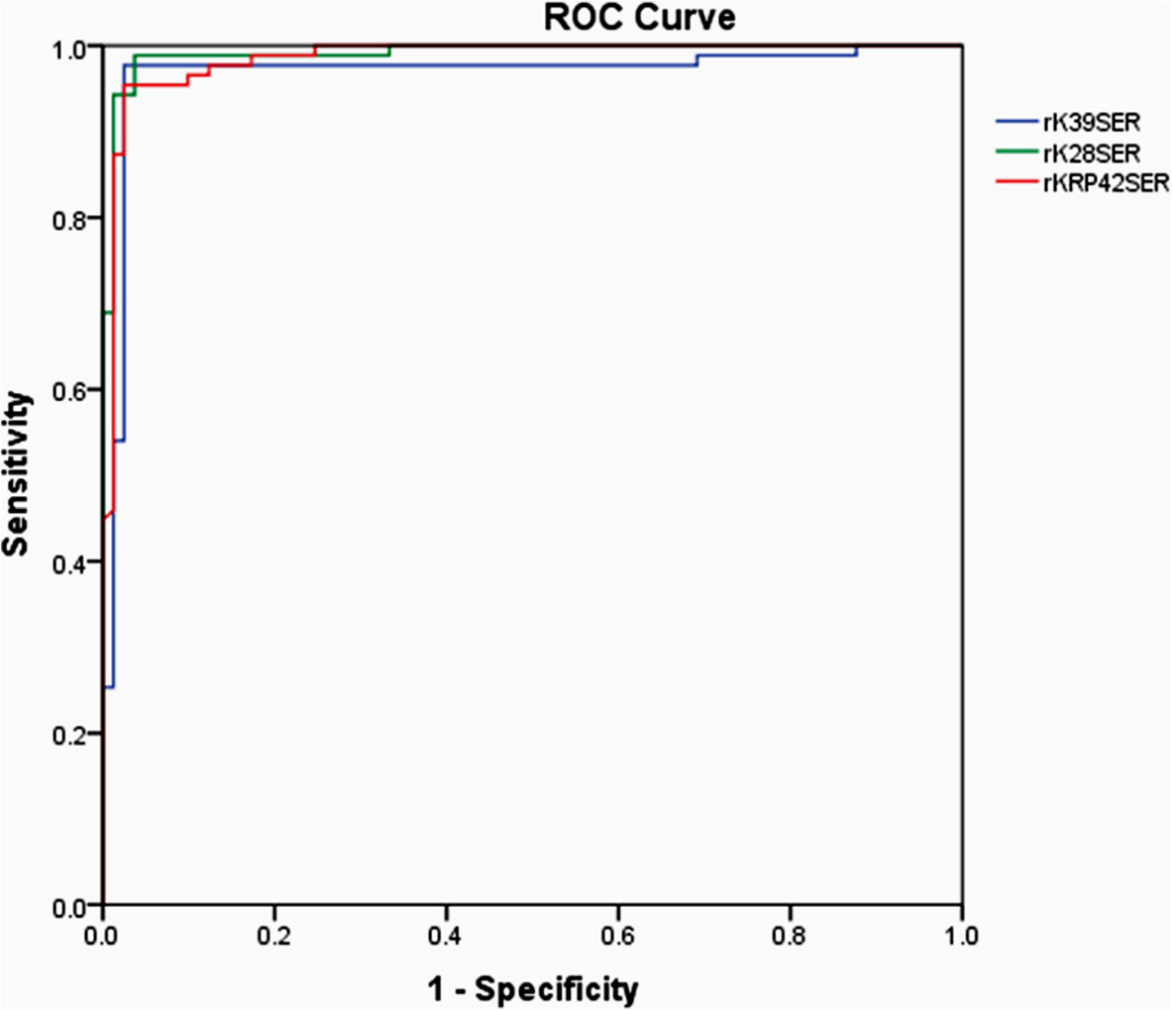
Multiple ROC curve generated from the OD (450 nm) values obtained after performing ELISA in serum

ELISA was performed using serum samples from 87 VL and 81 non-VL participants to detect anti-rK39, rK28 and rKRP42-IgG antibodies. The cut-off values for positivity and negativity were determined by ROC after analyzing the absorbance values at 450 nm (Fig. 1). The cut-off values of rK39, rK28 and rKRP42 were 2.379, 2.263 and 1.115 respectively. Based on the cut-off values (Table 2), rK28 ELISA showed the greatest sensitivity (98.9 %) with marginally lower sensitivity of rK39 and rKRP42 (97.7 and 95.4 %, respectively). Considering all of the control groups together, rK39 and rKRP42 showed equal specificity (97.5 %) while rK28 showed specificity of 96.3 % (Tables 3, 4 and 5). Among NEC, each of the antigens showed a 100 % specificity, but in the EC and DC groups, each of the antigens gave one false positive result. Among TB patients, rK39 and rKRP42 showed a 100 % specificity while rK28 showed 93.8 % specificity (Tables 3, 4 and 5). The values for area under the curve (rK39 = 0.968; rK28 = 0.991; rKRP42 = 0.985) for each of the antigens indicated a high degree of diagnostic accuracy, because the AUC value equal to 1 represents a 100 % accuracy (Table 2) [23]. Furthermore, the agreement (0.952 ≥ kappa value ≥ 0.881) was excellent among three ELISA methods (Table 6). Taken together, these data confirm that antibodies against of each of these antigens are abundant in the serum of VL patients.

**Table 2:** The values of AUC and cut-off OD of ELISA methods performed with rK39, rK28 and rKRP42 recombinant antigens

ELISA	Analyte	Cut-off OD	Area under the curve
rK39	Serum	2.37875	0.968
	Urine	0.19650	0.984
rK28	Serum	2.26250	0.991
	Urine	0.20250	0.987
rKP42	Serum	1.11450	0.985
	Urine	0.15550	0.970

**Table 3:** Sensitivity and specificity of rK39 ELISA performed using serum and urine samples from VL patients and non-VL individuals for diagnosis of VL

	Serum					Urine			
Group	Subjects (*n*)	Positive (*n*)	Sensitivity *n* (%)	Specificity *n* (%)	95 % CI	Positive (*n*)	Sensitivity *n* (%)	Specificity *n* (%)	95 % CI
VL	87	85	85 (97.70)	na	91.94–99.72	82	82 (94.25)	na	87.10–98.11
All controls (EC + NEC + DC + TB)	81	2	na	79 (97.53)	91.36–99.70	2	na	79 (97.53)	91.36–99.70
EC	33	1	na	32 (96.97)	84.24–99.92	1	na	32 (96.97)	84.24–99.92
DC	16	1	na	15 (93.75)	69.77–99.84	0	na	16 (100)	79.41–100
TB	16	0	na	16 (100)	79.41–100	1	na	15 (93.75)	69.77–99.84
NEC	16	0	na	16 (100)	79.41–100	0	na	16 (100)	79.41–100

*Abbreviations*: *na*, not applicable; 95 % CI, sensitivity or specificity at 95 % confidence interval

**Table 4:** Sensitivity and specificity of rK28 ELISA performed using serum and urine samples from VL patients and non-VL individuals for diagnosis of VL

	Serum					Urine			
Group	Subjects (*n*)	Positive (*n*)	Sensitivity N (%)	Specificity *n* (%)	95 % CI	Positive (*n*)	Sensitivity *n* (%)	Specificity *n* (%)	95 % CI
VL	87	86	86 (98.85)	na	93.76–99.97	83	83 (95.40)	na	88.64–98.73
All controls (EC + NEC + DC + TB)	81	3	na	78 (96.30)	89.56–99.23	3	na	78 (96.30)	89.56–99.23
EC	33	1	na	32 (96.97)	84.24–99.92	1	na	32 (96.97)	84.24–99.92
NEC	16	0	na	16 (100)	79.41–100	1	na	15 (93.75)	69.77–99.84
DC	16	1	na	15 (93.75)	69.77–99.84	0	na	16 (100)	79.41–100
TB	16	1	na	15 (93.75)	69.77–99.84	1	na	15 (93.75)	69.77–99.84

*Abbreviations*: *na*, not applicable; 95 % CI, sensitivity or specificity at 95 % confidence interval

**Table 5:** Sensitivity and specificity of rKRP42 ELISA performed using serum and urine samples from VL patients and non-VL individuals for diagnosis of VL

	Serum					Urine			
Group	Subjects (*n*)	Positive (*n*)	Sensitivity *n* (%)	Specificity *n* (%)	95 % CI	Positive (*n*)	Sensitivity *n* (%)	Specificity *n* (%)	95 % CI
VL	87	83	83 (95.40)	na	88.64–98.73	79	79 (90.80)	na	82.68–95.96
All controls (EC + NEC + DC + TB)	81	2	na	79 (97.53)	91.36–99.70	3	na	78 (96.30)	89.56–99.23
EC	33	1	na	32 (96.97)	84.24–99.92	0	na	33 (100)	89.42–100
NEC	16	0	na	16 (100)	79.41–100	1	na	15 (93.75)	69.77–99.84
DC	16	1	na	15 (93.75)	69.77–99.84	1	na	15 (93.75)	69.77–99.84
TB	16	0	na	16 (100)	79.41–100	1	na	15 (93.75)	69.77–99.84

*Abbreviations*: *na* not applicable; 95 % CI, sensitivity or specificity at 95 % confidence interval

**Table 6:** Agreement between different ELISA methods in serum and urine

	Kappa (serum)	Agreement	Kappa (urine)	Agreement
rK39 ELISA *vs* rK28 ELISA	0.952	Excellent	0.857	Excellent
rK39 ELISA *vs* rKRP42 ELISA	0.905	Excellent	0.786	Good
rK28 ELISA *vs* rKRP42 ELISA	0.881	Excellent	0.786	Good

### Diagnostic performance of rK39, rK28 and rKRP42 using urine

**Fig. 2:**
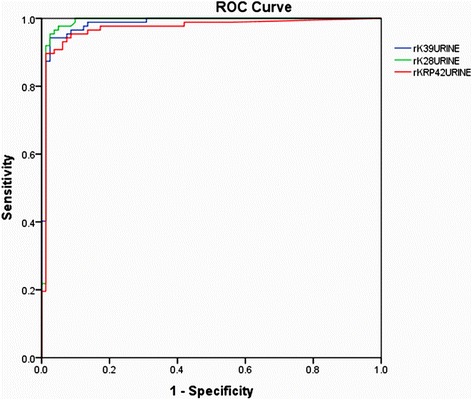
Multiple ROC curve generated from the OD (450 nm) values obtained after performing ELISA in urine

ELISA were conducted using urine as the test analyte, based on the cut-off values obtained from ROC curve (Fig. 2 and Table 2). The cut-off values of rK39, rK28 and rKRP42 were 0.197, 0.203 and 0.156, respectively. According to the cut-off values, rK39, rK28 and rKRP42 demonstrated 94.3, 95.4 and 90.8 % sensitivity, respectively. Of the three antigens, rK28 and rKRP42 showed equal specificity (96.3 %) and rK39 showed 97.5 % specificity when considered against all of the control groups (Tables 3, 4 and 5). In the EC group, rK39 and rK28 showed 97.0 % specificity while rKRP42 was indicated to be a 100 % specific. One false positive was found for both rK28 and rKRP42 antigens in the NEC group, but no NEC urine reacted with rK39. Further, in the DC urine, 100 % specificity was found for both rK39 and rK28, while rKRP42 showed specificity of 93.8 %. One false positive was found for each antigen in TB group. The values of area under curve (rK39 = 0.984; rK28 = 0.987; rKRP42 = 0.970) obtained from ROC curve analysis for three antigens to urine were comparable to those to serum (Table 2). Further, rK28 showed excellent agreement with rK39 (k = 0.857) whereas rKRP42 presented a good agreement (k = 0.786) with both rK39 and rK28 (Table 6). Thus, antibodies against each of these diagnostic antigens were readily and specifically detected in the urine of VL patients.

## Discussion

In this study, the diagnostic efficacy of rK28 ELISA has been determined using urine as an alternative, non-invasive diagnostic tool for VL. Also, the diagnostic performance of three antigen-specific antibody ELISA was evaluated and compared using urine and serum as the biological test sample. All of the antigens showed excellent diagnostic performance in serum. For rK39 which is the standard diagnostic antigen well used for VL, we observed 97.7 % sensitivity and 97.5 % specificity, corroborating the findings of a previous study performed in Bangladesh [24]. The sensitivity and specificity of rK39 ELISA on serum has varied from region to region [10, 11, 25], however, the rK28 antigen was developed to address this deficit [12, 22]. In this study we found 98.85 % sensitivity and 96.30 % specificity for rK28 ELISA developed with serum, performance levels consistent with previous studies [12, 22]. Similarly, our data generated in rKRP42 ELISA had sensitivity and specificity levels comparable with a previous study [14]. For each antigen, development of ELISA using urine rather than serum delivered similar sensitivities and specificities. Thus, our data reveal the diagnostic efficacy of rK28, rK39 and rKRP42 ELISA using urine as an alternative analyte that can be obtained by non-invasive means, suggesting suitability for broader use in VL-endemic regions and in individuals for whom serum collection is problematic.

The main limitation of the current study lies in the inclusion of only rK39 dipstick negative endemic controls. In addition, one study performed in Bangladesh reported cross-reactivity of malaria with VL and we did not include malaria cases within our control groups to determine ELISA specificities [21]. Thus, specificity of each ELISA might have been overestimated. Although the ELISA method is not well suited for field settings, for diagnosis purposes, urine samples could easily be collected and transported to centralized laboratories for analyses. Although, this study inherits several limitations, the promising diagnostic performance of rK28 ELISA in urine necessitates further evaluation of rK28 ELISA through prospective studies. In addition, several studies have reported excellent performance of serum in RDT incorporating the rK28 antigen and this RDT should be evaluated on urine [12, 26]. To the best of our knowledge, this is the first study on rK28 ELISA using urine samples.

The detection of antibodies in urine by rK39 and rK28 was achieved with sensitivities comparable to those observed with serum, whereas rKRP42 had comparatively poor sensitivity in urine. Several studies performed on rKRP42 ELISA and rK39 RDT reported comparable specificities with this study using urine [14, 17, 21, 27]. Compared to our data, one previous study found the sensitivity of rK39 ELISA to be poor (54.0 %) [28]. In that study, the poor performance of rK39 ELISA in urine was attributed to the inappropriate ELISA method and use of *L. chagasi*-derived coating antigen [28]. A previous study performed in Bangladesh reported a relatively higher sensitivity of rKRP42 ELISA than the data reported herein, although in that study the coating antigen concentration was double [14]. Therefore, the mild difference in sensitivity between the two studies might be attributed to the variance in ELISA methods. It is noteworthy that the cut-off OD values for anti-rKRP42 antibodies in both serum and urine also depict the relatively lower immune-reactivity of rKRP42 than observed with rK39 and rK28. Regardless, each of the antigens showed excellent specificity for detecting antibodies in urine.

All antigens have given sporadic false positives within the four control groups. However, in the EC and TB groups, the number of false positives was higher. For the EC and DC groups, the false positives could be potentially due to asymptomatic or latent *L. donovani* infection. Alternatively, many studies have reported cross-reactivity of VL with other infectious diseases (malaria, typhoid, leprosy and amebiasis) [8, 18], and in TB patients (notably collected from a non-endemic region) the false positive results could reflect actual cross-reactivity of VL with TB [8, 29]. The false positives in NEC might be due to the binding of unknown urinary components with rK39, rK28 and rKRP42 antigens [21]. Despite these rare false positive results, high specificities were observed for each ELISA developed with urine.

rK28 showed the highest sensitivity both in serum and urine in this study. Given that rK28 is a fusion poly-protein comprising regions of *L. donovani* haspb1 (*L. infantum* k26 homologue), *L. donovani* kinesin (*L. infantum* k39 homologue) and *L. donovani* haspb2 (*L. infantum* k9 homologue) the cumulative detection of antibodies against each of these components likely enhances sensitivty over individual antigens, particulalry rK39 [12]. It was comforting, but not surprising, that excellent agreement was found between rK28 and rK39 ELISA (k = 0.857; Table 6). rKRP42 ELISA also presented good agreement with rK28 ELISA in urine (k = 0.786; Table 6). The specificity of rK28 ELISA, both in serum and urine, was 96.3 % (95 % CI: 89.56–99.23). Thus, our findings substantiate that rK28 urine ELISA is a promising diagnostic tool for VL.

Since the kala-azar elimination programme is on target to reduce the rate of VL to 1 per 10,000 in endemic areas in the Indian sub-continent by 2015, a strategy of active case detection is going to be deployed to maintain the targeted case rate standstill by the programme which has extended its activity till 2017 [5, 7, 30]. According to the current strategy, rK39 RDT is the sole serodiagnostic tool while this method has several limitations [30, 31]. Our data indicate that rK28 urine ELISA has shown sensitivity and specificity consistent with the criteria that has been defined by Boelaert et al. [9] for use of diagnostic tools in active case detection. The potential for blood-borne transmission of HIV, hepatitis B virus (HBV) and hepatitis C virus (HCV) is high if blood is drawn through unsterilized needles/syringes, and the use of improper techniques can result in further complications [27]. A urine-based method can circumvent these concerns as it provides a truly non-invasive means of sample collection. Also, urinary antibody remains stable for a prolonged period at four degrees, permitting sample transfer for evaluation in a reference laboratory [20, 32]. Hence, rK28 urine ELISA could be considered as a potential alternative or adjunct to the present serological methods that are being used for diagnosis of VL.

## Conclusion

The findings of this study corroborate the potential of rK28 ELISA for diagnosis of VL by detecting antibody in urine. Considering the versatile advantages of urine as an alternative analyte of serum, rK28 urine ELISA could be considered as an alternative or adjunct to the present serological methods for diagnosis of visceral leishmaniasis.

## Abbreviations

DAT, Direct agglutination test; ELISA, Enzyme-linked immunosorbent assay; IFAT, Indirect fluorescent antibody test; KEP, Kala-azar elimination program; RDT: Rapid diagnostic test; VL, Visceral leishmaniasis.

## Additional file

Additional file 1: Table S1. Participants’ detail of disease control group. (DOCX 14.2 kb)
