# Supplementary material for: Evaluation of diagnostic performance of rK28 ELISA using urine for diagnosis of visceral leishmaniasis
Source: Parasit Vectors. 2016 Jul 4;9:383. doi: 10.1186/s13071-016-1667-2 (PMC4932727; doi:10.1186/s13071-016-1667-2)
Supplement: Additional file 1: Table S1. — Participants’ detail of disease control group. (DOCX 14.2 kb) [file 13071_2016_1667_MOESM1_ESM.docx]

| Type | Name of the Disease | Number of patients |
| --- | --- | --- |
| Disease control | Pyrexia of unknown origin | 3 |
| Disease control | Rheumatic fever | 1 |
| Disease control | Enteric fever | 2 |
| Disease control | Liver abciss | 1 |
| Disease control | Acute lymphoblastic leukemia | 1 |
| Disease control | Chronic liver disease | 2 |
| Disease control | Aplastic Anemia | 1 |
| Disease control | Thalasemia | 1 |
| Disease control | Chronic myeloblastic leukemia | 1 |
| Disease control | Viral hepatitis | 1 |
| Disease control | Acute myeloblastic leukemia | 1 |
| Disease control | Space occupying lesion in spleen | 1 |

Table 1: Participants’ detail of disease control group
